# Evaluation and Perception of the Immediate Impact of COVID-19 on Dental Education: An Observational Study

**DOI:** 10.7759/cureus.46979

**Published:** 2023-10-13

**Authors:** Tirth Bhutada, Surekha Dubey, Priyanka Paul, Jahnavi Gorripati

**Affiliations:** 1 Public Health Dentistry, Sharad Pawar Dental College, Datta Meghe Institute of Higher Education and Research, Wardha, IND; 2 Prosthodontics, Sharad Pawar Dental College, Datta Meghe Institute of Higher Education and Research, Wardha, IND

**Keywords:** dental teachers, dental students, online education, dental education, covid 19

## Abstract

Background

The primary aim of the study is to assess how the COVID-19 pandemic has affected dental education for students and teachers, particularly concerning the efficiency of online teaching strategies for both clinical and academic outcomes. Additionally, it also emphasizes psychological behavior like stress and anxiety in the post-pandemic era due to academic pressure.

Materials and methods

The study included 300 undergraduate dental students from various years of studying and 31 teachers actively teaching in dental college from all nine departments. The questionnaire was created in Google Forms for both students and teachers. The questionnaire contained various aspects of an education system that was adopted post-pandemic. The questions were given to evaluate the opinions of students as well as teachers to understand which method of teaching is preferable for them. Whether it's an online method of teaching or the offline mode, out of which the majority of respondents preferred the offline method of teaching.

Result

Most of the respondents agreed that the pandemic had affected their academic as well as clinical performance. Online mode of learning was not as effective as face-to-face learning. It did not affect the students clinically but mentally, which made them more anxious and stressed out during their studies and preparation for exams. Teachers too had to face a lot of problems from technical aspects while teaching online which led to mental stress and anxiety. This study re-emphasizes the negative impact of COVID-19 on dental education. It also further highlights the more fundamental changes and improvements that are desperately needed in the education system to adapt and overcome the current pandemic situation and future such events.

Conclusion

The COVID-19 pandemic has significantly impacted educational institutions, curricula, and clinical practice. Dental students are particularly vulnerable, and online education platforms in India face challenges in regulation. The quality of materials, technical assistance, and expertise in online classes significantly impact teaching and learning experiences. The pandemic has led to concerns about students and staff contracting the disease, creating a skill gap in dental graduates, and loss of clinical exposure, causing more anxiety. To address these concerns, regular, open, and compassionate communication with students is essential. Educational institutions must undergo a transformation to adapt to the pandemic and potential future disasters.

## Introduction

Dental schools provide challenging, competitive learning environments. They differ greatly from medical education in terms of clinical training and instruction in critical and scientific thinking. The field of dentistry requires both intellectual and technical abilities, both of which are dependent on training in the fundamental sciences that are clinically applicable and training in clinical care that is grounded in science. In India to prepare students for licensing and to begin practicing after graduation, dental schools have a two year preclinical and two year clinical sciences curriculum [1]. Because of the COVID-19 pandemic, eye-to-eye homeroom instructive exercises with undergrad and postgraduate students were almost intruded on around the world. The pandemic circumstance of COVID-19 affected clinical instruction internationally prompting the dropping of talks, research facility works out, clinical postings, and tests. To go on with the scholarly program, web-based classes have begun in various scholastic streams in enormous scope. This accompanied difficulties and undertakings to take on some changes for students and teachers [2].

Numerous dental schools across India had suspended clinical practices except in emergencies, simultaneously others were managing with the help of social distancing in the preclinical laboratory. Considering the time, manpower, and technology, simulation of mannequins is often difficult for accessing online teaching methods [3]. Starting from a daily physical mode of teaching, hands-on laboratory work, and clinical training all of them were halted by the respected Dental colleges, they have been using computer-based tests, virtual workshops, problem-solving sessions, and multimedia online education to make up for this. Preclinical and clinical training is crucial for students in dentistry, and COVID-19 had the worst negative impact on this [4].

The fundamental point of dental schooling is to train dental students to become independent and treat patients carefully. It has been demonstrated that student improvement in hand-eye coordination, fine motor skills, and introspective capacities are especially beneficial in the early stages of skill learning, resulting in a more cautious approach to planning and improved skill retention. Given the limited time and resources available, it becomes challenging to teach these skills at the necessary level. In virtual mode, this type of instruction is problematic [4].

For assessments and appraisals, the lockdown has driven dental schools to sort out assessments altogether on the web, delay developmental tasks, or concede summative evaluations. This led to a lot of problems in the evaluation of students' knowledge gained in online sessions and its application. This unnecessarily increases the period of academic sessions. It should be noted that during this crisis, due to the high viral exposure to frontline professionals involved in illness incidences and deaths, mainly doctors and dentistry students were concerned that they might be severely affected, possibly leading to depression. As a result, following the COVID-19 epidemic, the demand for counseling therapy and psychiatric assistance should increase [4].

## Materials and methods

After obtaining ethical approval from the Institutional Ethics Committee (DMIHER(DU)/IEC/2023/1189), Sharad Pawar Dental College and Hospital, Datta Meghe Institute of Higher Education and Research (DMIHER), Wardha. A focused group contained two faculty members and an undergraduate student who were effectively associated with dental training for piloting the study. A questionnaire was distributed among 31 faculty members and 300 undergraduates. Feedback was collected according to the clarity and sequence of the questions and whether the questions covered matched the study's objectives. The medium used to forward the survey was Google Forms. The link was shipped off to dental students in their respective clinical years at the Sharad Pawar Dental College, Sawangi, Wardha by means of e-learning accounts. One more modified link was to be shipped off to teaching faculty who regulate clinical meetings during the time of COVID-19 flare-up. A subsequent reminder update was sent in the following two and five days. The study was over in a short span of 30 days from July 1, 2022, to July 30, 2022. 

This survey contains a few inquiries that explored the effects of the COVID-19 pandemic on an educational system from dental students' and teachers' perspectives. The requirement for submission and recording of responses was to respond to all multiple-choice questions. This overview investigated the following few angles of concern the effect of COVID-19 on theoretical lectures, the effects of the pandemic on students’ performance and flow of patients, effects on the clinical exposure, the impact on academic performance and clinical competence, the effects of COVID 19 on stress, anxiety, and educational pressure.

Inclusion criteria

Students

All undergraduate students who enrolled at the college before the commencement of the COVID-19 pandemic, or prior to the 2019-20 academic year. Students who were exposed to both offline and online education techniques.

Teachers

Teachers who joined the college before the COVID-19 pandemic started or prior to the 2019-20 academic year. Teachers who taught both online as well as offline lectures.

Exclusion criteria

Students

All postgraduate students. Students who joined the college after the physical mode of teaching was resumed.

Teachers

Teachers who joined the college after the physical teaching mode was resumed.

## Results

The online study was done to understand the experience of the immediate impact of COVID-19 on dental education for students and teachers. The questionnaires along with all the responses were entered on Google Sheets. The percentages of a response collected and the needful results were analyzed. The study results are divided into two sections, one for students and the other for teachers.

Students

The responses were received from 300 undergraduate students. The responses received were from first year n=43 (14.3%), second year n=73 (24.3%), third year n=63 (21%), final year n=53 (17.7%), and interns n= 68 (22.7%) (Table 1).

**Table 1:** Total responses from dental students (n=300)

Variables (Years in Dental College)	n (%) (From 300 responses)
1st	43 (14.3%)
2nd	73 (24.3%)
3rd	63 (21%)
Final Year	53 (17.7%)
Intern	68 (22.7%)

The majority of students n=273 (91%) preferred the offline method of teaching over the online mode after experiencing both methods of teaching in the post-pandemic era. Only a few numbers n=27 (9%) preferred the online mode of teaching over the offline mode. Among all students, n=259 (86.3%) felt that the offline method was more effective in understanding as compared to the online method. Only n=41 (13.7%) found out online method better to understand. Pandemic n=243 (81%) students felt that their academic performance had a negative impact and only n=57 (19%) felt there was no negative impact on their academic performances, whereas n=242 (80.7%) hurt their clinical performance and only n=58 (19.3%) had no negative impact on their clinical performances, which overall reflected a lot during the examination period. The patient flow was adversely affected according to n=263 (87.7%) of students post-pandemic whereas only n=37 (12.3%) felt there was no disruption in patient flow. This led to the incompletion of their patient quota of n=192 (64%) students. Post-pandemic n=130 (43.3%) numbers of students felt that they were not able to see various variety of patients in their clinical posting. Total n=155 (51.7%) students were not satisfied with the clinical exposure they experienced post-pandemic area considering fewer numbers of the patient count. COVID-19 not only affected the students clinically and academically but also mentally. The majority of students n=222 (74%) were feeling stress related to studies. Only a few students n=78 (26%) felt no stress during the pandemic period. Not only that n=217 (72.3%) felt more pressure than normal during the COVID period for their studies. This led to n=214 (71.3%) students feeling more anxious than normal while preparing for their examinations (Table 2).

**Table 2:** Student's responses

Questions	Result			
Which method of learning do you prefer?	Online	n=27 (9%)	Offline	n=273 (91%)
Are online lectures more effective in understanding than offline?	Yes	n=41 (13.7%)	No	n=259 (86.3%)
Do you think COVID-19 has affected your academic performance in a negative way at a certain level?	Yes	n=243 (81%)	No	n=57 (19%)
Do you think COVID-19 has affected your clinical performance in a negative way at a certain level?	Yes	n=242 (80.7%)	No	n=58 (19.3%)
Has the pandemic affected patient flow?	Yes	n=263 (87.7%)	No	n=37 (12.3%)
Are you able to complete your patient quota?	Yes	n=108 (36%)	No	n=192 (64%)
Post-pandemic, are you satisfied with your clinical exposure?	Yes	n=145 (48.3%)	No	n=155 (51.7%)
Post-pandemic, have you seen the various variety of patients?	Yes	n=170 (56.7%)	No	n=130 (43.3%)
During the lockdown period, was any kind of stress related to studies bothering you?	Yes	n=222 (74%)	No	n=78 (26%)
Post-pandemic, do you feel more pressure for studies?	Yes	n=217 (72.3%)	No	n=83 (27.7%)
Do you feel more anxious while preparing for exams post-COVID-19?	Yes	n=214 (71.3%)	No	n=86 (28.7%)

Teachers

A total of 31 teachers responded to the Google Forms questionnaire mentioned above. Out of which n=5 (16.12%) responses were from Oral Medicine and Radiology, n=4 (12.9%) responses from Conservative Dentistry and Endodontics, n=4 (12.9%) responses from Oral Maxillofacial Surgery, n=4 (12.9%) responses from Periodontics, n=4 (12.9%) responses from Prosthodontics, n=3 (9.6%) responses from Oral Pathology and Microbiology, n=3 (9.6%) responses from Orthodontics, n=2 (6.5%) responses from Public Health Dentistry, and n=2 (6.5%) responses from Pedodontics (Table 3).

**Table 3:** Total responses of teachers (n=31)

Variables (Departments in Dental College)	n (%) (From 31 responses)
Oral Medicine and Radiology	5 (16.12%)
Conservative Dentistry and Endodontics	4 (12.9%)
Oral Maxillofacial Surgery	4 (12.9%)
Periodontics	4 (12.9%)
Prosthodontics	4 (12.9%)
Orthodontics	3 (9.6%)
Oral Pathology	3 (9.6%)
Pedodontics	2 (6.5%)
Public Health Dentistry	2 (6.5%)

Out of 31 responses, n=31 (100%) every teacher who participated in the study felt the offline mode of teaching was preferable as compared to the online mode. Adapting suddenly to the online method of teaching wasn't easy for n=19 (61.3%) number of respondents compared to n=12 (38.7%) felt the other way. Being a teacher n=27 (87.1%) respondents are not satisfied with the method of virtual teaching with no physical presence. Also, n=27 (87.1%) number of respondents were not satisfied with teacher-student interaction during online classes. Only n=4 (12.9%)were satisfied with the student-teacher interaction. The majority of respondents n=20 (64.5%) faced problems in adapting from physical mode to virtual mode as the whole dimension of the teaching got changed. The main problem teachers faced frequently where the technical glitches occurring during the online lectures. A total of n=23 (74.2%) of teachers were not confident in solving technical glitches during online teaching. Taking attendance in virtual mode was not easy as n=16 (51.6%) number of teachers found it difficult to record their attendance. COVID-19 took away a big chunk of time from academic sessions, making it difficult for teachers to complete the syllabus. The only option to complete the syllabus was via online lecture, out of which the majority n=19 (61.3%) teachers found it difficult to complete the syllabus in online mode. Compared to only n=12 (38.7%) were able to complete the syllabus in time. The most important thing for any teacher is the satisfaction they get after delivering the lecture to students, it was found that n=28 (90.3%) teachers were not satisfied with their delivery of lectures online, and only n=3 (9.7%) teachers were satisfied with their delivery of lectures (Table 4).

**Table 4:** Teacher's responses

Questions	Result			
Which method do you prefer to teach students?	Online	n=0 (0%)	Offline	n=31 (100%)
Was the online method of teaching easy to adapt and execute?	Yes	n=12 (38.7%)	No	n=19 (61.3%)
Were you satisfied with the teacher-student interaction during online classes?	Yes	n=4 (12.9%)	No	n=27 (87.1%)
Being a teacher, were you satisfied with the method of virtual teaching with no physical presence?	Yes	n=4 (12.9%)	No	n=27 (87.1%)
Was changing from a physical mode of teaching to a virtual mode a smooth experience?	Yes	n=11 (35.5%)	No	n=20 (64.5%)
Do you feel confident in solving technical glitches during online teaching?	Yes	n=8 (25.8%)	No	n=23 (74.2%)
Was taking attendance in online mode an easy task for you?	Yes	n=15 (48.4%)	No	n=16 (51.6%)
Was the syllabus completion via online mode easy for you?	Yes	n=12 (38.7%)	No	n=19 (61.3%)
Did the delivery of the lecture online satisfy you as a teacher?	Yes	n=3 (9.7%)	No	n=28 (90.3%)

## Discussion

In this observational study, we evaluated dental students' and faculty's perceptions of the immediate impact of COVID-19 on dental education. COVID-19 has hit our education system quite hard. A lot of challenges were faced, not only by the students but also from the perspective of teachers. The result of the study indicates that the majority of the students as well as teachers preferred the offline method of teaching over online anytime. Michal Baczek et al. reported that there was no statistical difference between face-to-face and online learning in terms of the ability of the learning method to increase knowledge but they discovered that when it came to improving skills and social competency, e-learning was deemed to be less effective than face-to-face instruction. In comparison to regular classes, they also discovered that online classes had fewer active students [5]. In this study, both teachers and students felt that understanding the topic through a physical mode of the lecture is way easier as compared to online lectures. Similarly, O'Malley and McCraw found that students initially believed they couldn't learn as much online as they could in face-to-face courses when they signed up for online classes [6]. During the pandemic, a new mode of teaching that is online virtual lecture was introduced. Shifting from physical mode to virtual mode was not an easy task for teachers. They experienced a lot of technical glitches as this medium of teaching was very new for the teachers. Taking attendance to interact with students was not a smooth experience for a teacher. Yang and Cornelius found a similar conclusion in their study [7]. They discovered that students experience course dissatisfaction when instructors are unable to offer technical support. Teachers also face difficulty in completing the syllabus on time.

Post-pandemic it was found that most of the students felt that their academic preparation had been severely affected negatively. Son et al. also found the same finding which showed that due to the long-lasting pandemic situation and onerous measures such as lockdown and stay-at-home orders, the COVID-19 pandemic hurts higher education [8]. It not only affected academics but a major impact was found in clinical performances as well, which led to various problems in developing clinical skills. Jumah et al. in their study found that the COVID-19-related disruption of academic and clinical activity has had an unavoidable professional impact on the incoming generation of dental graduates [9]. Another reason for the decline in clinical performance was the lack of patient flow. Post-pandemic, the patient variety, and even the number have declined. COVID-19 not only affected the students and teachers physically but also mentally. The majority of students felt more stress and anxiety about their studies and exams post-pandemic. Choi et al. in their study showed the same outcome, demonstrating that student assistantship interruptions had the most impact on students' readiness and confidence [10]. Also, Akinkugbe et al. concluded in their study that academic dental institutions must take responsibility for the students' increased anxiety levels and offer appropriate training and support to lessen its impact [1]. Teachers were also under pressure and were anxious due to various technical problems faced during online lectures. The main aim of any lecture from a teacher's perspective is the level of satisfaction they get after delivering a lecture. But knowing from this study, teachers were not satisfied with their delivery of lectures online.

This study highlighted COVID-19's detrimental effects on dentistry education once more. Additionally, it emphasizes how urgently the educational system has to evolve and develop to cope with the current pandemic emergency and any such catastrophes in the future. Deepika Nambiar conducted a study regarding teachers' and student's perceptions and experiences related to virtual online classes [11]. The findings reveal that timely and effective student-professor communication, the availability of technical support, carefully designed online courses, and adjustments to enable the delivery of practical lessons are all essential for ensuring that teachers and students are satisfied with online learning [11].

Limitations of the study

The study had certain limitations. This study was conducted only on dental graduates, not on medical graduates, so their perspective is still not known. The study was done in a limited Geographic area that did not relate wider population. Also, this study was done after the pandemic was over so there are still many aspects that affected the students and teachers during the COVID-19 pandemic.

## Conclusions

The COVID-19 pandemic and its effect on educational institutions, curricula, and clinical practice are without a doubt unprecedented. This study shows that dental students are vulnerable to COVID-19's effects. Lack of clarity on the regulation of online education platforms now limits higher education in India. The quality and quantity of the study materials, technical assistance, and general expertise with online class delivery all have a major impact on the overall teaching and learning experience. They also have an impact on how much contact there is between students and faculty. These elements ultimately determine whether online education is successful or not. To promote the use of online channels by students and teachers, sensitivity is required, with a focus on the criteria of convenience and accessibility. In the early phases of the pandemic, worries about students and staff members contracting and spreading the disease were expressed. There may be an unavoidable skill gap in the forthcoming class of dental graduates as a result of the significant disruption to this student cohort's academic and clinical activities. There is a lot of pressure on teachers to adjust to the unexpected shifts in how they teach, to find solutions for skill shortages, and to ensure safe clinical practice. This study shows how the dental education system needs to improve its curriculum to concentrate more intently on methods for practicing in the "new normal." To address concerns about an adaptable and enlarged curriculum, regular, open, and compassionate contact with students is necessary. This may help to reduce some of the tension and anxiety they feel. Our educational institutions and approaches urgently require a transformation to take into account the effects of the current pandemic and potential future occurrences of comparable disasters.
